# Use of mulberry–soybean intercropping in salt–alkali soil impacts the diversity of the soil bacterial community

**DOI:** 10.1111/1751-7915.12342

**Published:** 2016-02-19

**Authors:** Xin Li, Minglong Sun, Huihui Zhang, Nan Xu, Guangyu Sun

**Affiliations:** ^1^ College of Life ScienceNortheast Forestry UniversityHarbin150040China; ^2^ Key Laboratory of Forestry Plant Ecology of Ministry of EducationNortheast Forestry UniversityHarbin150040China; ^3^ College of Resources and EnvironmentNortheast Agricultural UniversityHarbinHeilongjiang Province150030China; ^4^ Natural Resources and Ecology InstituteHeilongjiang Academy of SciencesHarbin150040China

## Abstract

Diverse intercropping system has been used to control disease and improve productivity in the field. In this research, the bacterial communities in salt–alkali soils of monoculture and intercropping mulberry and soybean were studied using 454‐pyrosequencing of the 16S rDNA gene. The dominant taxonomic groups were *Proteobacteria*,* Acidobacteria*,* Actinobacteria*,* Chloroflexi*,* Bacteroidetes*,* Planctomycetes* and *Gemmatimonadetes* and these were present across all samples. However, the diversity and composition of bacterial communities varied between monoculture and intercropping samples. The estimated bacterial diversity (H') was higher with intercropping soybean than in monoculture soybean, whereas H' showed an opposite pattern in monoculture and intercropping mulberry. Populations of *Actinobacteria*,* Acidobacteria*, and *Proteobacteria* were variable, depending on growth of plants as monoculture or intercropped. Most of *Actinobacteria* and *Chloroflexi* were found in intercropping samples, while *Acidobacteria* and *Proteobacteria* were present at a higher percentage in monoculture samples. The plant diversity of aboveground and microbial diversity of belowground was linked and soil pH seemed to influence the bacterial community. Finally, the specific plant species was the major factor that determined the bacterial community in the salt–alkali soils.

## Introduction

Alkalization and salinization of soils the two largest factors limiting global agricultural productivity (Liu *et al*., 2010). Alkalization and salinization are especially problematic in the Songneng Plain in China which has 3.2 × 10^6^ ha salt‐affected land and is one of the three largest soda saline‐alkali areas in the world. Every year, about 20 × 10^3^ ha land becomes salinized/alkalized (Wang *et al*., 2009). Saline soils negatively impact plant growth due to osmotic inhibition of water absorption (Munns, 2002) and an excess of sodium ions that affects critical biochemical processes. The pernicious effects of alkalinity on plant growth are related to high pH, which disrupts ion balance (Shi and Zhao, 1997) and poor micronutrient availability in soil (Alam *et al*., 1999). Therefore, salt or alkali soils can tremendously negatively affect productivity of both natural grasslands and crop plants grown on cultivated lands. The productive capacity of alkaline soils could be improved by growing plants adapted to sodic soils (Gupta *et al*., 1990). In addition, soil organic matter content and availability of soil inorganic nitrogen can be improved via reclamation agroforestry (Singh *et al*., 1997).

Intercropping has been used for many years to grow two or more crop species together simultaneously (Willey, 1979). Compared with monoculture, intercropping often leads to greater stability of yields (Wiley, 1979), resilience to perturbations (Trenbath, 1999) and reduced N‐leaching (Hauggaard‐Nielsen *et al*., 2003). Use of a tree/N‐fixing crop intercropping system can be an especially sustainable and beneficial agricultural practice, as the N‐fixing crop provides natural N fertilizer for growing trees. Nitrogen‐fixing legumes may even be improved by intercropping (Neumann *et al*., 2007), because the intercropped cereals or trees can be more competitive for nitrogen in soil, forcing the legume crop to fix more atmospheric N_2_ (Hauggaard‐Nielsen *et al*., 2003). In addition, legume/cereal mixtures, grown in pots, together achieved greater P uptake from soils (El Dessougi *et al*., 2003) than can either species could take up by itself. In the field, increased P uptake by intercropped maize/faba bean also was observed (Li *et al*., 2003).

In saline and alkaline soils, excessive amounts of salts have an adverse effect on biological activity including soil enzyme activity (Rao and Pathak, 1996), nitrogen mineralization (McClung and Frankenberger, 1985) and soil microbial biomass (Tripathi *et al*., 2006). Soil microbial communities are important in managed field systems, as they are involved in most nutrient transformations in soil and thereby influence the aboveground plant productivity (Van Der Heijden *et al*., 2008). Soil microbial communities are greatly influenced by salinity (Rietz and Haynes, 2003), as well as other abiotic and biotic environmental variables (Alexander, 1977). In alkaline soils, the biological activity of microbial communities improves, when a crop, grass or tree cover is used (Rao and Ghai, 1985).

Soil is a complex and dynamic habitat for microorganisms, which are involved in many key process required for ecosystem functionality (Lim *et al*., 2010). The diversity of microorganisms in soils can often exceed that, found in other environments (Rosselló‐Mora and Amann, 2001) and is higher by orders of magnitude than the biodiversity of plants and animals. In order to better understand the changes of bacterial communities in monoculture versus intercropped plants (mulberry and soybean) in salt–alkali soils, a 16S rDNA gene‐based pyrosequencing approach was employed using field‐grown mulberry/soybean intercropped under salt–alkali conditions. To our knowledge, this is the first report on the changes of bacterial communities of mulberry/soybean intercropping under salt–alkali stress conditions.

## Results

Pyrosequencing analysis of the V1‐V3 region of the 16S rDNA genes resulted in 71 660 high‐quality sequence reads with a read length of ≥ 400 bp across all four samples. The average read length was 482 bp. The read numbers were uneven per sample, ranging from 17 193 to 18 819, with an average of 17 915 (Table S1). We were able to classify 65 926 (92%) of the quality sequences below the domain level. As fewer sequences result in lower numbers for predicted operational taxonomic units (OTUs) and species richness (Will *et al*., 2010), all samples were randomly reduced to the same size using MOTHUR, based on the sample with the smallest number of reads.

### Bacterial diversity and richness

**Figure 1:**
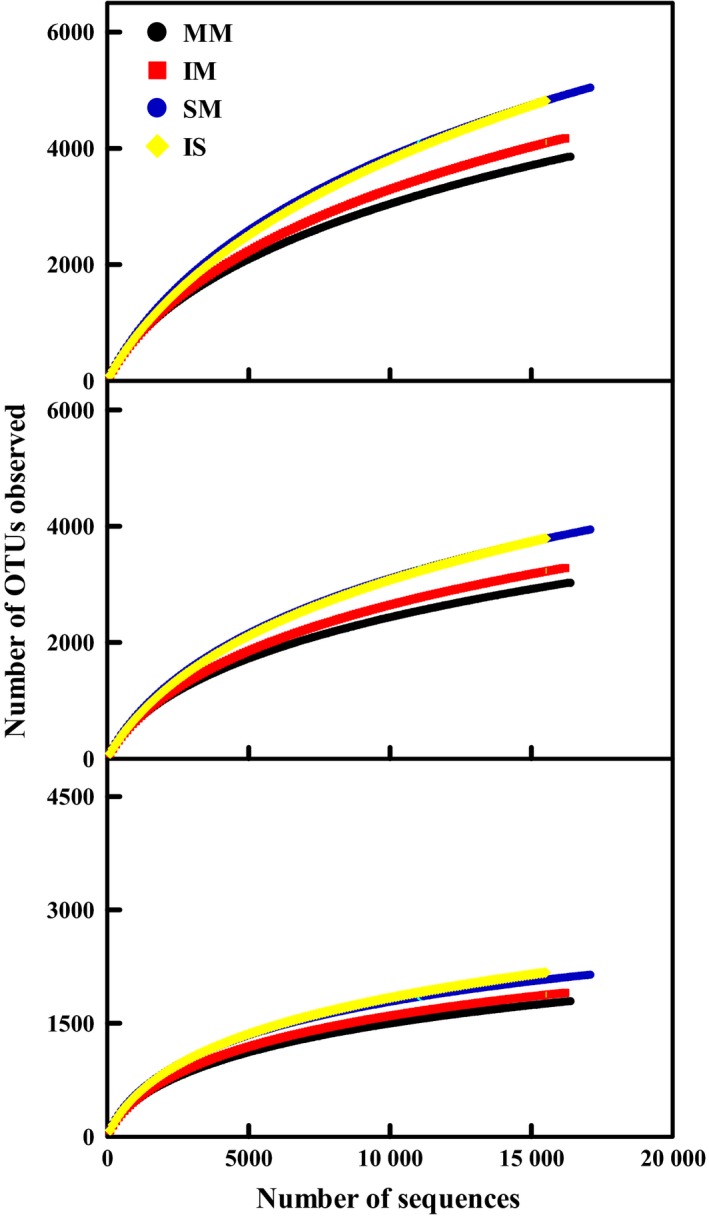
Rarefaction curves indicating the observed number of OTUs at genetic distances of 3%, 5% and 10% in all soil samples. MM, mulberry monoculture; IM, intercropping mulberry; SM, soybean monoculture; IS, intercropping soybean.

To determine rarefaction curves and other measures of diversity, OTUs were identified at genetic distances of 3%, 5% and 10%. Rarefaction curves indicated consistent differences in all four samples (Fig. 1). At 10% genetic distances, almost all rarefaction curves reached saturation, indicating that the surveying effort covered almost the full extent of taxonomic diversity at this genetic distance. At 3% and 5% genetic distances, rarefaction curves suggested that the sequencing effort was not large enough to capture the complete diversity of these communities, as the curves did not level off with increasing sample size. The comparison of mean Chao 1 richness estimates of mulberry rhizosphere soils and with soybean rhizosphere soils showed different values at genetic distances of 3% (6609 OTUs and 8296 OTUs respectively), 5% (4980 OTUs and 6083 OTUs respectively) and 10% (2552 OTUs and 2990 OTUs respectively), and the richness was higher in soybean rhizosphere soils (*P* < 0.05) (Table 1). Analysis of differences of ways of planting by employing Student's *t*‐test at genetic distances of 3%, 5% and 10% showed that the intercropping patterns did not vary significantly in the predicted number of OTUs (*P* > 0.05). The same conclusion was seen using the Ace richness index (Table 1). Moreover, comparison of the mean Shannon diversity index of all samples revealed that the highest bacterial diversity at all analysed genetic distances was found in SM, followed by IS, IM and MM. The predicted richness and diversity in the soybean monoculture rhizosphere soils exceeded that of the corresponding mulberry monoculture soils. Thus, an influence of plant species on bacterial richness and diversity was observed. Intercropping impacted overall bacterial diversity.

**Table 1:** Bacterial diversity as assessed by Shannon index (H′) and species richness estimation in all soils

Genetic distance (%)	Sample	H′	Chao1	ACE
			No. operational taxonomic units	
3	MM	7.40 ± 0.016a	6407 ± 266.36a	7739 ± 213.15a
	IM	7.71 ± 0.021b	6810 ± 260.62a	8156 ± 214.78a
	SM	7.88 ± 0.016a	8070 ± 270.39a	9694 ± 229.47a
	IS	7.75 ± 0.021a	8522 ± 328.80a	10735 ± 276.82a
5	MM	7.10 ± 0.016a	4883 ± 226.78a	5545 ± 167.02a
	IM	7.38 ± 0.016b	5076 ± 207.96a	5000 ± 157.25a
	SM	7.51 ± 0.021a	5942 ± 212.02a	5911 ± 165.00a
	IS	7.39 ± 0.021a	6223 ± 255.32a	7236 ± 196.01a
10	MM	6.23 ± 0.021a	2526 ± 126.78a	2441 ± 88.28a
	IM	6.53 ± 0.021b	2578 ± 112.48a	2560 ± 86.23a
	SM	6.66 ± 0.016a	2847 ± 115.32a	2774 ± 81.74a
	IS	6.59 ± 0.021a	3124 ± 145.14a	3049 ± 105.02a

Different letters following the mean values within each column indicates significant differences at *P* < 0.05.

MM, mulberry monoculture; IM, intercropping mulberry; SM, soybean monoculture; IS, intercropping soybean.

### Distribution of taxa and phylotypes across all samples

**Figure 2:**
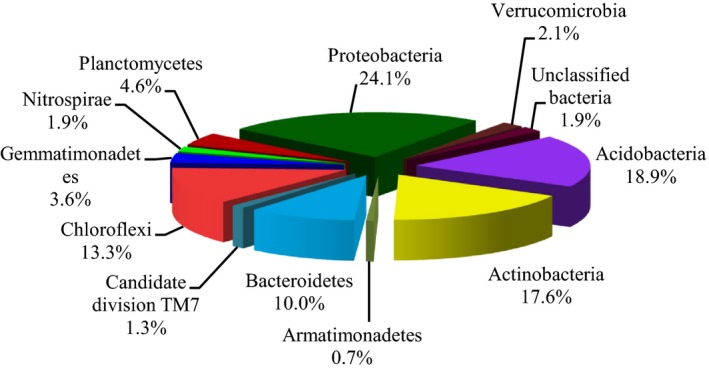
Proportional distribution of different phyla. MM, mulberry monoculture; IM, intercropping mulberry; SM, soybean monoculture; IS, intercropping soybean.

**Figure 3:**
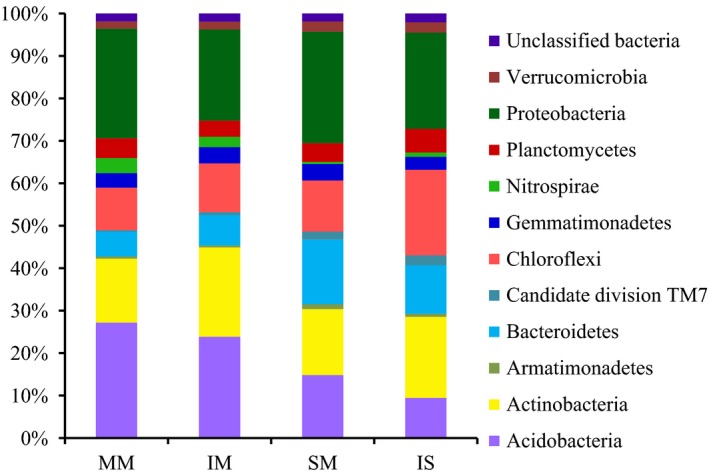
Relative abundance of bacterial phyla for each soil library. MM, mulberry monoculture; IM, intercropping mulberry; SM, soybean monoculture; IS, intercropping soybean.

The 65 926 classifiable sequences were affiliated with 11 bacterial phyla (Fig. 2). The groups accounted for 96% of all sequences, and a few sequences (< 1%) could not be shown (e.g. *Spirochaetes*). The dominant phyla across all samples were *Proteobacteria*,* Acidobacteria*,* Actinobacteria*,* Chloroflexi*,* Bacteroidetes*,* Planctomycetes* and *Gemmatimonadetes*, representing 24.1%, 18.9%, 17.6%, 13.3%, 10.0%, 4.6% and 3.6%, respectively, of all sequences that were classified below the domain level. These dominant bacterial phyla were found in all samples (Fig. 3). Other sequences belonged to *Verrucomicrobia*,* Nitrospirae*,* Armatimonadetes* and unclassified bacteria of ‘*Candidatedivision* TM7’, and they were always found in very low proportions (< 2%).

Comparative analysis of the four soil samples revealed a distinct distribution of the bacterial phyla (Fig. 3). On average, *Chloroflexi* and *Bacteroidetes* showed a higher relative abundance in soybean rhizosphere soils than in mulberry rhizosphere soils, whereas *Acidobacteria* and *Nitrospirae* showed the opposite pattern. The phyla of *Actinobacteria*,* Acidobacteria*,* Proteobacteria* and *Chloroflexi* were found in variable proportions, depending on the use of monoculture or intercropping; most of *Actinobacteria* and *Chloroflexi* were found in intercropping samples, whereas *Acidobacteria* and *Proteobacteria* were present at higher percentages in monoculture samples.

At the classes' level, the bacterial community composition also revealed distinction between different soils (Table 2). Within the *Acidobacteria*, all samples were dominated by *Acidobacteria* and *Holophagae*, and significant differences were observed between monoculture (MM and SM) and intercropping (IM and IS) soils (*P* < 0.05). Furthermore, members of *Chloroflexi* phylum showed a opposite pattern to the one observed for *Anaerolineae*, which *Anaerolineae* shows a higher abundance in intercropping libraries than monoculture libraries. In comparison to MM libraries, the IM libraries showed a decrease in the OTUs number of *Deltaproteobacteria*, while other classes of *Proteobacteria* showed no significant differences. Within in *Bacteroidetes*, all samples were dominated by *Sphingobacteria*, and SM libraries had more OTUs number than IS libraries (*P* < 0.05). In constrast, the *Planctomycetes*,* Actinobacteria* and *Gemmatimonadetes* were present in similar proportions in monoculture and intercropping samples.

**Table 2:**
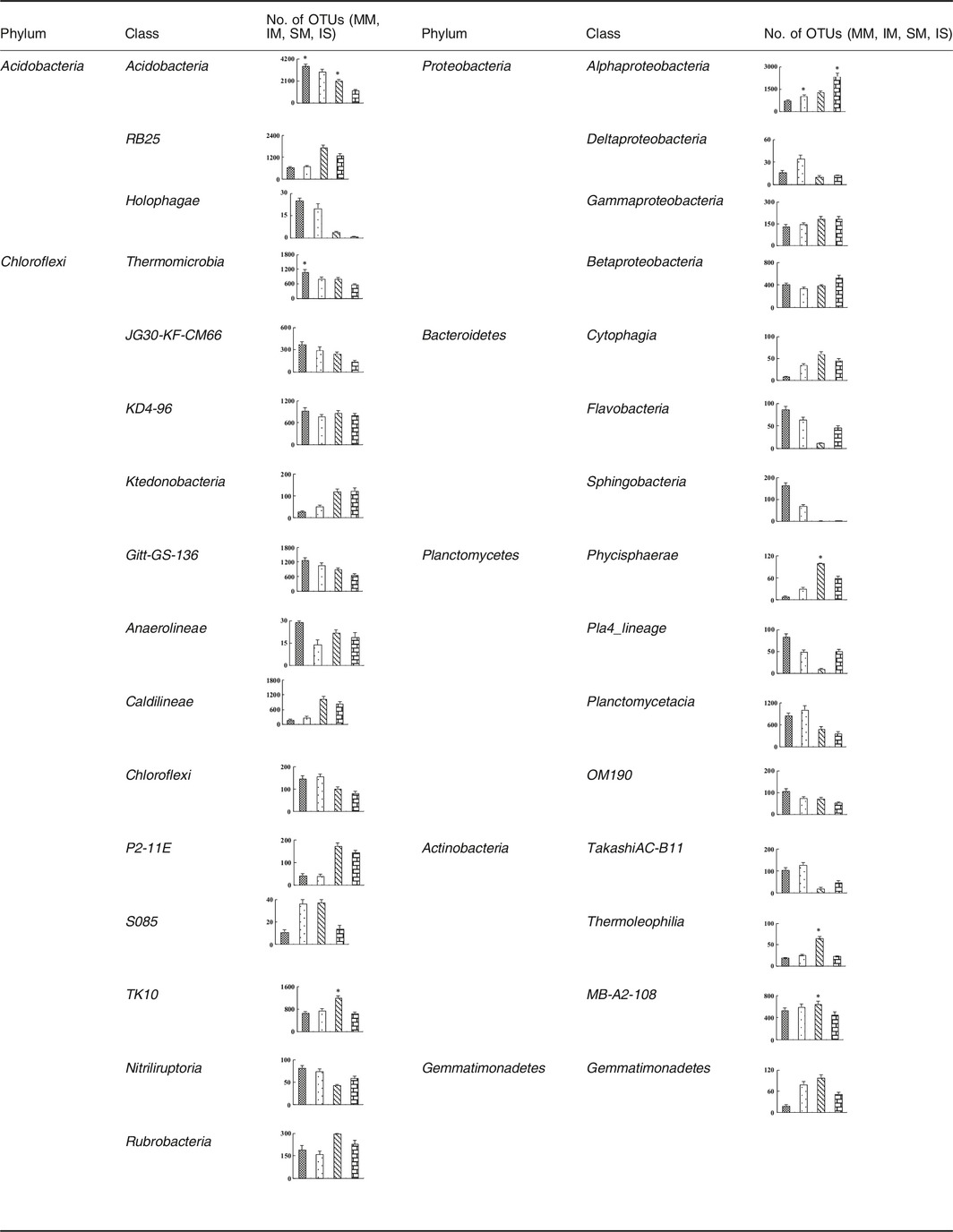
Taxonomic classification of bacterial reads retrieved from four samples at phylum and class levels from 16S rDNA gene pyrosequencing

### Shared bacterial OTUs

**Figure 4:**
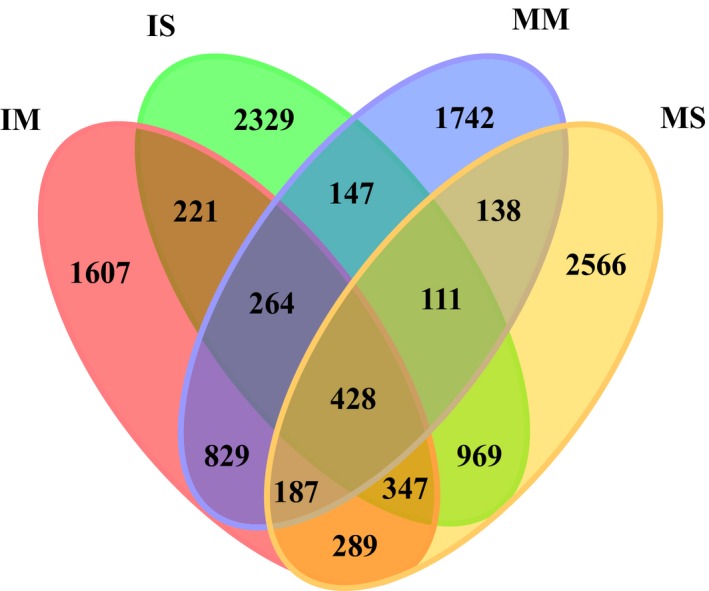
Venn diagram showing the shared bacterial OTUs (at a distance of 0.03) in all soil samples. MM, mulberry monoculture; IM, intercropping mulberry; SM, soybean monoculture; IS, intercropping soybean.

Venn diagrams revealed that the sum of total observed OTUs in the four soil samples was 12 174 (Fig. 4), and 417 OTUs were common all of the soil samples. Moreover, the distribution of sequences demonstrated once again that each plant rhizosphere had its own microbial population.

**Figure 5:**
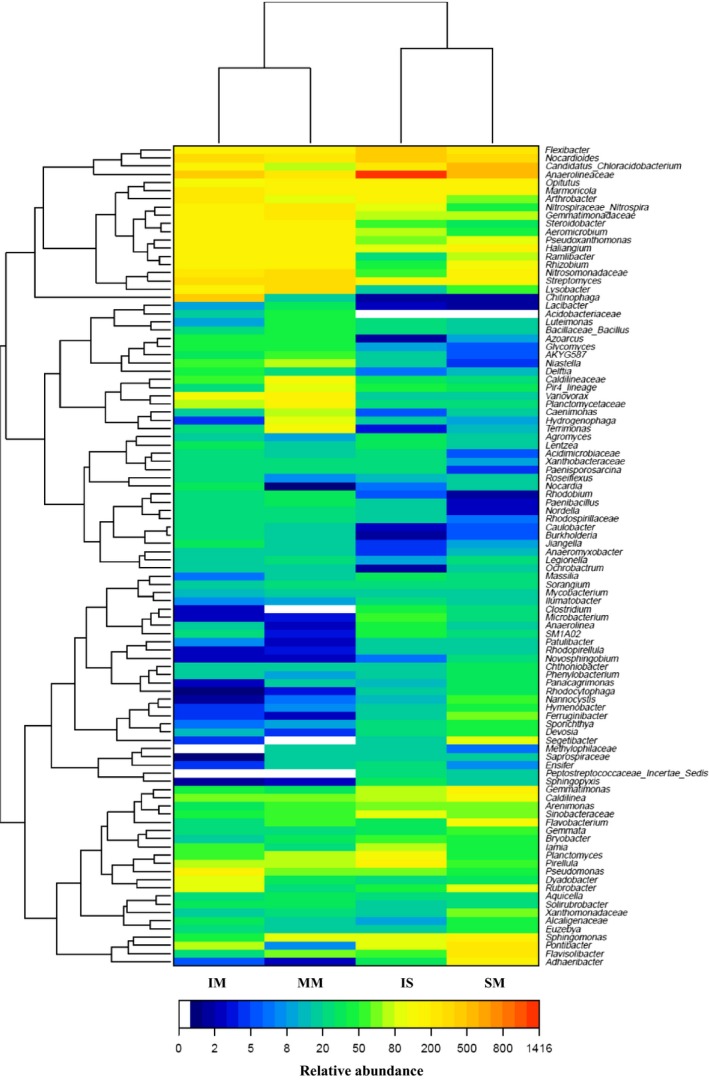
Hierarchical cluster analysis of 100 predominant bacterial communities among the four samples. The *Y*‐axis is the clustering of the most abundant OTUs (5% distance) in reads. The OTUs were ordered by genus. Sample communities were clustered based on complete linkage method. The colour intensity of scale indicates relative abundance of each OTU read. Relative abundance was defined as the number of sequences affiliated with that OTU divided by the total number of sequences per sample.

**Figure 6:**
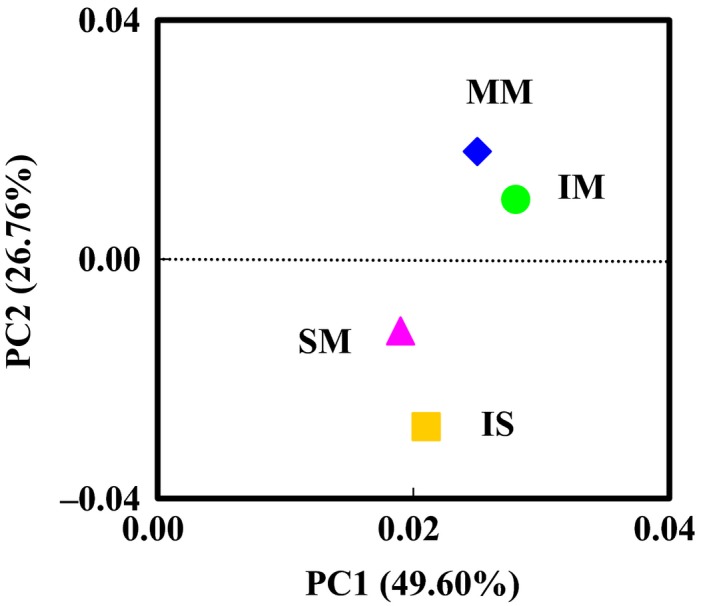
Principal component analysis (PCA) of bacterial communities from mulberry monoculture (MM), intercropping mulberry (IM), soybean monoculture (SM), and intercropping soybean (IS) based on pyrosequencing of the 16S rDNA gene. PCA were generated using the presence of each OUT (at a distance level of 3%) found in each clone library. Principal components (PCs) 1 and 2 explained 49.60% and 26.76% of the variance respectively.

Hierarchically clustered heatmap analysis, based on the microbial community profiles at the genus level, was used to identify the different composition of these four microbial community structures (Fig. 5). The MM and IM groups were separated from SM and IS groups, suggesting clear distinctions of microbial community structure between mulberry and soybean groups. This was supported by the principal component analysis (PCA) with the weighted Unifrac distance (Fig. 6). Overall, the two PCA axes explained 77.36% of the variation between the different communities. The PCA score plot revealed that the mulberry and soybean rhizosphere soils harboured characteristic bacterial communities. Mulberry samples (MM and IM) were clustered together and were well separated from that soybean samples (SM and IS), whereas there was a little distinction between SM and IM samples. These results suggested that plant species had the greatest affect on the bacterial communities in the soil used to support those plants.

At the genus level, comparison of the relative abundances revealed significant differences between monoculture and intercropping soil bacterial communities (Fig. 5). *Anaerolinea*,* Nocardioides*,* Candidatus*_*Chloracidobacterium*,* Flexibacter*,* Streptomyces* and *Nitrosomonadaceae* were the most abundance genera across all soil samples, representing 3.44%, 2.71%, 3.03%, 1.81%, 1.59% and 1.81% of all classified sequences in monoculture soils and 8.75%, 3.22%, 1.87%, 2.71%, 1.93% and 1.19% in intercropping soils. This indicated these genera might be indigenous in the salinized meadow soil sampled. The distribution of the dominant genera varied significantly between monoculture and intercropping soils. *Anaerolineaceae* (2.95%), *Nocardioides* (2.15%), *Candidatus_Chloracidobacterium* (1.05%), *Arthrobacter* (1.43%), *Pontibacter* (0.52%), *Chitinophaga* (3.02%), *Pseudomonas* (1.02%), *Rubrobacter* (0.64%) and *Nocardia* (0.62%) showed a higher relative abundance in mulberry intercropping soils than in corresponding monoculture soils, whereas *Pir4_lineage* (0.54%), *Terrimonas* (0.56%), *Hydrogenophaga* (0.49%), *Caenimonas* (0.48%), *Acidobacteriaceae* (0.27%) and *Lacibacter* (0.24%) showed the opposite pattern (Fig. 5). *Candidatus_Chloracidobacterium* (5.36%), *Nitrosomonadaceae* (1.09%), *Flavisolibacter* (2.70%), *Pontibacter* (2.00%), *Ramlibacter* (0.74%), *Adhaeribacter* (1.53%), *Flavobacterium* (0.85%), *Xanthomonadaceae* (0.60%), *Segetibacter* (0.79%), *Alcaligenaceae* (0.38%) and *Ferruginibacter* (0.58%) were present in higher proportions in soybean monoculture soils compared with corresponding intercropping soils (Fig. 5).

### Correlations of environmental data and bacterial communities

**Figure 7:**
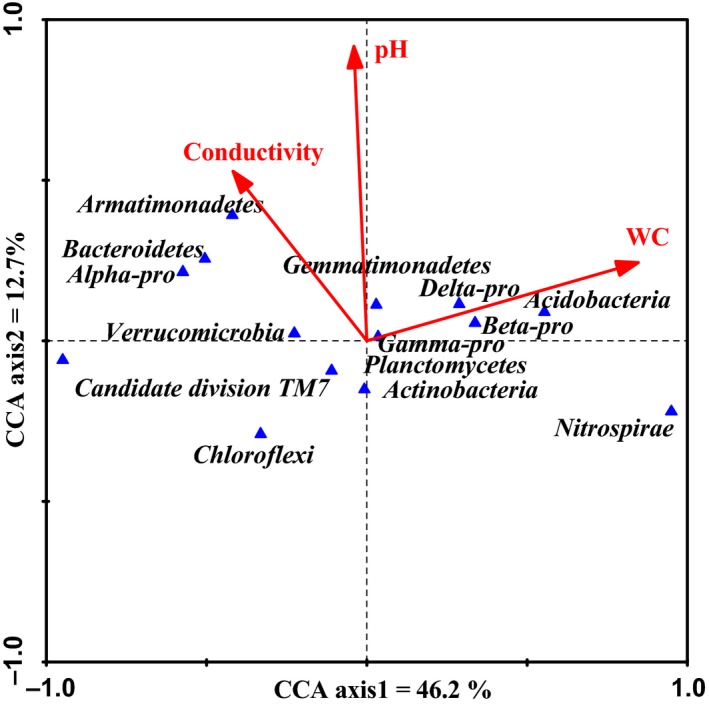
Canonical correspondence analysis (CCA) for soil bacterial community and soil environmental variables. Arrows indicated the direction and magnitude of measurable variables associated with bacterial community structures. Each triangle represents a different bacterial phyla or proteobacterial class.

To investigate relationships between soil microbial community composition and measured environmental variables, different bacterial phyla and proteobacterial classes were analysed using canonical correspondence analysis (CCA) (Fig. 7). The influence of the environmental parameter on the CCA bioplot is indicated by arrows in which lengths are proportional to their importance (Liu *et al*., 2009). pH had the longest arrow, indicating that it was most important in influencing the bacterial community. Conductivity was also significantly linked to bacterial phyla variance in CCA. With the exception of *Nitrospirae*, the relative abundances of all of bacterial phyla and classes positively correlated with soil pH and conductivity. Water content (WC) was also negatively correlated with *Chloroflexi*,* Candidatedivision* TM7 and *Planctomycetes*.

## Discussion

Our previous studied have indicated, with an intercropped soybean/mulberry crop, the aboveground biomass of intercropped mulberry increased 65.7% compared with monoculture mulberry during the co‐growing stage (Zheng *et al*., 2011). The beneficial effect may be attributed to the improvement of soil nutrient element and the reduction in pH (Li *et al*., 2007), as well as microbial activity and community composition in intercropping systems (Bastida *et al*., 2008).

Using 454‐pyrsequencing, we assessed bacterial communities' composition and diversity in salt–alkali soils of monoculture and intercropping mulberry and soybean collected from Songnen Plain of China. In this study, for microbial analysis of soil, the dominant taxonomic groups were *Proteobacteria*,* Acidobacteria*,* Actinobacteria*,* Chloroflexi*,* Bacteroidetes* and *Planctomycetes* (Fig. 2). These phyla have been described as common inhabitants of non‐saline–alkali soils (Fierer *et al*., 2012; Kuramae *et al*., 2012). Shannon diversity analyses revealed a richer bacterial community in intercropping mulberry soil than that of monoculture soil (Table 1). This observation may be supported by previous studies which demonstrate that the bacterial communities of intercropping mulberry have higher diverse than monoculture. The presence of soybean plants contributed to attenuate eventual bacterial community variations occurring in intercropping mulberry. Indeed, tree‐based intercropping systems present a more heterogeneous vegetation cover, a patchier distribution of plant litter and rooting patterns that can affect soil properties and microbial communities (Reynolds *et al*., 2007; Lacombe *et al*., 2009).

The bacterial community structure in soybean rhizosphere was influenced from that of the communities in mulberry rhizosphere (Fig. 3). Several studies indicated that the interactions among plant species, growth stages and soil types could influence microbial communities in rhizosphere soil (Marschner *et al*., 2001; Wieland *et al*., 2001; Song *et al*., 2007). In the rhizosphere of soybean, the bacterial community structure in rhizosphere soils of monoculture and intercropping were different. Most of *Actinobacteria* and *Chloroflexi* were found in intercropping samples, whereas *Acidobacteria* and *Proteobacteria* were present at higher percentages in monoculture samples. The high similarities were found in monoculture and intercropping mulberry, indicating that an important integrated role of intercropping in shaping the soil microbial communities structure. *Actinobacteria* have a critical role in decomposition of soil organic materials, such as cellulose and chitin (Sykes and Skinner, 1973). *Actinobacteria* of intercropping soils were higher than that of monocropped soils, which may be due to the presence of more organic matter used by plants in intercropping soils. Comparing to control soil (pH 9.14), the pH of soil of monoculture and intercropping soybean was significantly decreased to 7.30 and 7.60 (Table S2), respectively, which may be due to legume as P‐mobilizing plants species can release protons in alkaline soils (Li *et al*., 2014). The abundance of the phylum *Acidobacteria* correlates with the soil pH (Hartman *et al*., 2008; Jones *et al*., 2009) and acidobacterial subgroups 1 and 2 decreased with the increase in pH (Lauber *et al*., 2009), which is in contrast to our study. In our study, pH of intercropping mulberry (pH 7.32) were lower than that of monoculture soils (pH 7.73) (Table S2). The pH reduction in intercropping mulberry may be due to intercropped with legume. However, monoculture mulberry soils contained more *Acidobacteria* than the corresponding intercropped soils. *Acidobacteria* are capable of degradation of plant litter in soils (Eichorst *et al*., 2011), the presence of mulberry debris in monoculture samples and soybean root exudates and litter in intercropping samples may have contributed to the observed differences.

To investigate relationships between soil microbial community composition and measured environmental variables, different bacterial phyla and proteobacterial classes were analysed using CCA (Fig. 7). The pH and conductivity seemed to affect soil bacterial communities. Soil property such as pH and conductivity are expected to control microbial community composition (Drenovsky *et al*., 2004). Hence, a greater spatial heterogeneity of microbial communities was also expected in intercropping systems of salt–alkali soils. Fierer and Jackson (2006) demonstrated that pH influenced the overall diversity and composition of microbial communities in a range of terrestrial and aquatic environments. Salinity could also affect bacterial composition and diversity across a variety of habitats (Wakelin *et al*., 2012).

The recent studies of overyielding in agriculture intercropping systems found a important mechanism underlying such facilitation is the ability of some crop species to chemically mobilize otherwise‐unavailable forms of one or more limiting soil nutrients such as phosphorus (Li *et al*., 2014). Plants do not take up organic P directly; rather, organic P is first hydrolysed by microbial or root‐related phosphatases (Li *et al*., 2014). Therefore, phosphate‐solubilizing bacteria have an important role in soils with low concentration of available phosphorus. Phosphate‐solubilizing bacteria were present in different proportions in monoculture and intercropping soils in our study. *Burkholderia*,* Pseudomonas* and *Arthrobacter* showed more abundance in intercropping soils than that monoculture soils. These bacteria can improve solubilization of fixed soil phosphorus and applied phosphates resulting in higher crop yields (Nautiyal, 1999).

Legumes and non‐legumes can ‘complement’ each other in the use of N sources as both the legume and non‐legume utilize soil inorganic N sources, but nodule in leguminous plants can also fix atmospheric N_2_ in symbiosis with *Rhizobium* (Jensen, 1996). IS libraries had low *Rhizobium* than SM libraries (Fig. 5). However, most studies showed a opposite results that N_2_ fixation of legumes may be improved by intercropping when the non‐legume is a strong competitor for soil inorganic N (Giller *et al*., 1991; Karpenstein‐Machan and Stuelpnagel, 2000; Hauggaard‐Nielsen *et al*., 2001). The integration of trees with crops on moderately alkali soils improved soil total nitrogen and carbon status (Kaur *et al*., 2000), due to organic matter inputs in the form of litter fall and fine roots from trees, which showed plant‐soil feedback process are also important.

Plants release enormous chemicals through their roots, at a significant carbon cost, to combat pathogenic microorganisms and attract beneficial ones (Badri *et al*., 2009). The activity and effects of beneficial rhizosphere microorganisms on plant growth and health are well documented for bacteria like by *Pseudomonas* and *Burkholderia* (Badri *et al*., 2009). *Pseudomonas* showed a higher relative abundance in intercropping mulberry and soybean respectively. Similar as *Pseudomonales*,* Burkholderia* was present at higher relative abundance in intercropping mulberry. These bacteria protect several major agricultural crops against disease phenomenon that is likely to be also important in natural ecosystems (Van Der Heijden *et al*., 2008).

Principal component analysis clearly demonstrated that the bacterial communities were separated into mulberry and soybean groups, suggesting that plant species was the major factor in determining the bacterial community in the salt–alkali soils (Fig. 5). The distribution of sequences demonstrated that each plant rhizosphere had its own microbial population (Fig. 4), which indicated the appearance of some new microbial population were stimulated by plant diversity. Previous studies also observed that soil carry‐over influences of plant diversity are mediated by a general stimulated of soil microorganisms (Bartelt‐Ryser *et al*., 2005). Changes in relative abundance of different OTUs also demonstrated the important effects of intercropping.

In conclusion, in intercropped plants growing in salt–alkali soils, the diversity and composition of bacterial communities varied between monoculture and intercropping samples and some of beneficial plant rhizobacteria (phosphate‐solubilizing bacteria) are more abundant in intercropping samples. Soil pH under intercropping was significantly diminished in relation to control soil. Therefore, mulberry–soybean intercropping in the perspective of biology is good strategy to improve salt–alkali soils.

## Materials and Methods

### Field plots

The field site used in this research was located at the Yuejin village, Zhaozhou County of Heilongjiang province of Songnen Plain (latitude, 45° 70′ N; longitude, 125° 27′ E, northeast China). The average annual temperature in the region is 3.37°C and the mean temperature in July is 22.4°C. The mean annual precipitation is 418.9 mm and nearly 73.1% of total rainfall is received by northwest monsoons from July to September. The mean annual elevation is 1597.1 mm, annual sunshine hours are 3014.4 h, the active accumulated temperature (≥ 10°C) is 2921.3°C per year, and a frost‐free period of 137 days. The soil is classified as a salinized meadow soil, with a pH of 9.04, a salinity content of 0.37%. Selected soil properties were as follows: pH 9.14; salinity content 0.37%; Cl^−^, 0.23 mol kg^−1^; CO_3_^2−^, 0.09 mol kg^−1^; HCO_3_^−^, 0.47 mol kg^−1^; SO_4_^2−^, 0.16 mol kg^−1^; Na^+^, 0.11 mol kg^−1^; K^+^, 2.15 mol kg^−1^; Ca^2+^, 0.48 mol kg^−1^ and Mg^2+^, 0.15 mol kg^−1^.

The experimental design consisted of three blocks. Each block was divided into three plots representing the three plantation systems. Each plot unit comprised 12 rows that were 5 m long and 0.66 m wide, each 39.6 m^2^ in size. Plots and blocks were separated from each other by 1 m walkways. Treatments levels included (1) soybean monoculture, (2) mulberry monoculture and (3) soybean intercropped with mulberry. For the intercropped treatment, two soybean rows were intercropped with two rows of mulberry. The single cropping plots consisted of 12 rows of one plant species. Edges of each plot were sown with a mix of soybean and mulberry to minimize edge effects but these plants were not included in the harvest.

The grafted mulberry (root stocks of ‘*Qiuyu*’ cultivar with the scion of ‘*Tieba*’ cultivar) was planted on 10 May 2011 using approximately 30 cm seedlings at the spacing of 0.20 m within the rows. The soybeans (*Glycine max* cv. Heinong 34) were sown manually, and seedlings in each row were thinned after emergence to leave a density of 30 plants m^−2^. Prior to sowing soybeans, fertilizer in the form of organic manure (30 000 kg hm^−2^) and (NH_4_)_2_HPO_4_ (150 kg hm^−2^) were applied and the soil was disked to a depth of 10 cm. A conventional herbicide treatment was applied to the soybean at the 2–3 trifoliate leaf stages.

Soil samples were collected from all plots on 5 August 2011, when the soybean was showing early pod formation (R3 stage). Rhizosphere soils, adhering to the roots (Nazih *et al*., 2001), were collected by shaking the soil off the roots. Part of the sample was used for DNA extraction for determination of microbial populations while part of the sample was extracted for soil property analyses. For DNA extraction, soil samples were immediately placed into sterile centrifuge tubes under ice, transferred to laboratory within 24 h and kept frozen at −80°C until DNA extraction. For soil property analyses, soil samples were taken to the laboratory, and vegetation, roots and stones were removed prior to soil sieving (< 2 mm). Soil pH (soil/H_2_O ratio of 1:2) was measured using a pH meter with a glass electrode. Salinity was measured by determining the electrical conductivity (EC, dS m^−1^) of soil water extract (water to soil ratio, 2.5:1, vol:wt) using a conductivity meter. The soil WC was measured by weighting fresh soil samples. The samples were then dried in the oven at 105°C to a constant mass. Oven dry weight was then determined. Gravimetric soil WC was calculated as (wet soil weight‐dry soil weight)/dry soil weight.

### DNA extraction, amplification of 16S rDNA genes and pyrosequencing

Total microbial community DNA was isolated from 1 g of soil per sample using an Omega Bio‐Tek E.Z.N.A. Soil DNA extraction kit (Omega Bio‐Tek, Atlanta, GA, USA) according to the manufacturer's instructions. The extracted DNA was examined following electrophoresis in 1% agarose gel and the DNA was normalized to the same concentration prior to amplification. A ~455 bp region of the 16S rDNA gene, covering the V1‐V3 region was selected to construct the community library through tag pyrosequencing. The V1‐V3 region was amplified with universal primers 8F (5′‐AGAGTTTGATCCTGGCTCAG‐3′) and 533R (5′‐TTACCGCGGCTGCTGGCAC‐3′) containing the A and B sequencing adaptors (454 Life Sciences, Shanghai, China). For each sample, three independent amplification reactions were performed. The PCR mixture (final volume, 50 μl) contained 5 μM of each primer, ~5 ng of template DNA, 5× FastPfu PCR buffer and 2.5 U of FastPfu DNA Polymerase (MBI Fermentas, USA). The amplification conditions consisted of an initial denaturation at 95°C for 2 min and 25 cycles of denaturation at 95°C at 30 s, annealing at 55°C for 30 s, and extension at 72°C for 30 s, followed by a final extension period at 72°C for 5 min. During amplification, negative control reactions lacking template DNA were also performed to check for experimental contamination. The amplicons were then purified once by gel electrophoresis/isolation and an additional two times using the Wizard SV Geland PCR Clean‐Up System (Promega, Madison, WI, USA). Pyrosequencing was then carried on a Roche massively parallel 454 GS‐FlX sequencer according to standard protocols.

### Sequence analysis

Pyrosequencing flowgrams were converted to sequence reads using MOTHUR software (http://www.mothur.org) and analysed. The acquired sequences were filtered by evaluating data quality and removing primers and barcodes. Sequence were filtered by: (1) selecting sequences which contained barcode and forward primer and eliminate sequences with even a single base pair, (2) removing sequences shorter than 150 bp, with ambiguous base pairs, or with more than two wrong matches in primer and (3) eliminating barcodes and forward primers. After filtering, the effective sequences were clustered to OTUs based on phylum, class and genus levels using MOTHUR (Sun *et al*., 2009).

### Microbial community analysis

In order to divide OTUs, optimized sequences were reduced to 350 bp length and compared using silva108. Rarefaction curves based on identified OTU, species richness estimator Chao 1 were generated with MOTHUR for each sample (Chao, A. 1984). Taxonomic assignment of sequences was done using the Ribosomal Database Project classifier (minimum confidence of 80%). After phylogenetic allocation of the sequences to the phylum and genus levels, the relative abundance of a given phylogenetic group was set as the number of sequences per sample. Venn diagrams of unique and OTUs (0.03 cut‐off value) were drawn in order to highlight similarities and shared sequences between the different analysed samples. Hierarchical cluster (Heatmap) analyses were generated in MOTHUR using the gplots package of R. UniFrac, which described the similarity and difference between the four samples.

### Statistical analysis

All experimental data were processed by Microsoft EXCEL 2010. Data were analysed using one‐way analysis of variance ANOVA. Values shown are the means of three replicates ± SD.

## Supporting information


**Table S1.** Number of 16S rDNA gene sequences derived from all soil samples. Mulberry monoculture (MM), intercropping mulberry (IM), soybean monoculture (SM) and intercropping soybean (IS).
**Table S2.** Soil property from all soil samples. Mulberry monoculture (MM), intercropping mulberry (IM), soybean monoculture (SM) and intercropping soybean (IS). Different letters following the mean values within each column indicates significant differences at *P* < 0.05.
