# Supplementary material for: Use of mulberry–soybean intercropping in salt–alkali soil impacts the diversity of the soil bacterial community
Source: Microb Biotechnol. 2016 Feb 19;9(3):293–304. doi: 10.1111/1751-7915.12342 (PMC4835568; doi:10.1111/1751-7915.12342)
Supplement: Supplementary file 1 — Table S1. Number of 16S rDNA gene sequences derived from all soil samples. Mulberry monoculture (MM), intercropping mulberry (IM), soybean monoculture (SM) and intercropping soybean (IS). Table S2. Soil property from all soil samples. Mulberry monoculture (MM), intercropping mulberry (IM), soybean monoculture (SM) and intercropping soybean (IS). Different letters following the mean values within each column indicates significant differences at P < 0.05. [file MBT2-9-293-s001.docx]

**Table S1.**

| **Sample** | **No. obtained sequences ≥400 bp** | **No. sequences classified below domain level** |
| --- | --- | --- |
| MM | 17873 | 16338 |
| IM | 17775 | 16152 |
| SM | 18819 | 17098 |
| IS | 17193 | 16338 |

**Table S2**

| **Sample** | **pH** | **Conductivity (µs·cm^-1^)** | **Water content (%)** |
| --- | --- | --- | --- |
| MM | 7.73±0.02 b | 242.33±7.37 b | 15.34±2.31 a |
| IM | 7.32±0.03 a | 223.00±5.29 a | 13.62±3.68 a |
| SM | 7.30±0.03 a | 247.00±1.00 a | 12.42±2.98 a |
| IS | 7.60±0.03 b | 239.67±8.39 a | 13.87±3.11 a |
